# Analytical Solution to the Flory–Huggins Model

**DOI:** 10.1021/acs.jpclett.2c01986

**Published:** 2022-08-17

**Authors:** Daoyuan Qian, Thomas C. T. Michaels, Tuomas P. J. Knowles

**Affiliations:** † Centre for Misfolding Diseases, Yusuf Hamied Department of Chemistry, University of Cambridge, Lensfield Road, Cambridge, CB2 1EW, U.K.; ‡ Department of Physics and Astronomy, Institute for the Physics of Living Systems, University College London, London, WC1E 6BT, U.K.; § Laboratory for Molecular Cell Biology, University College London, London, WC1E 6BT, U.K.; ⊥ Cavendish Laboratory, Department of Physics, University of Cambridge, J J Thomson Avenue, Cambridge, CB3 0HE, U.K.

## Abstract

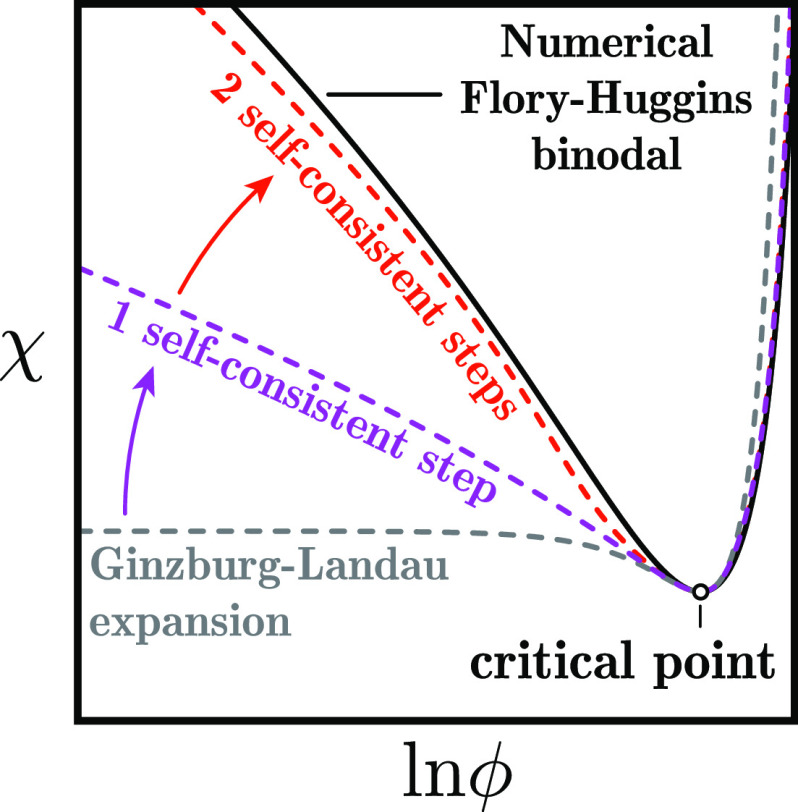

A self-consistent analytical solution for binodal concentrations of the two-component Flory–Huggins phase separation model is derived. We show that this form extends the validity of the Ginzburg–Landau expansion away from the critical point to cover the whole phase space. Furthermore, this analytical solution reveals an exponential scaling law of the dilute phase binodal concentration as a function of the interaction strength and chain length. We demonstrate explicitly the power of this approach by fitting experimental protein liquid–liquid phase separation boundaries to determine the effective chain length and solute–solvent interaction energies. Moreover, we demonstrate that this strategy allows us to resolve differences in interaction energy contributions of individual amino acids. This analytical framework can serve as a new way to decode the protein sequence grammar for liquid–liquid phase separation.

The formation of membrane-less organelles through liquid–liquid phase separation (LLPS) has emerged as an important mechanism used by cells to regulate their internal biochemical environments, and it is also closely related to the development of neurodegenerative diseases.^1−8^ The Flory–Huggins model^9−11^ is a foundational theoretical picture that describes the phenomenology of LLPS, driven by a competition of entropy and interaction energy. Despite the generality of the Flory–Huggins model, analytical solutions describing the binodal line have not been available, and progress has instead been made through numerical methods.^11−13^ Here, we propose an analytical self-consistent form for the binodal concentrations and demonstrate the high accuracy comparable to numerical schemes. This can then be used to efficiently fit experimental binodal data and determine key underlying physical parameters.

The two-component Flory–Huggins theory describes mixing of a polymer species of length *N* with a homogeneous solvent. Denoting the volume fraction of polymers as ϕ, the volume fraction of the solvent is simply 1 – ϕ by volume conservation. The model uses an effective lattice site contact energy  between the polymer and solvent in which *z* is a coordination constant, and ϵ_ps_, ϵ_ss_, and ϵ_pp_ are bare polymer–solvent, solvent–solvent. and polymer–polymer contact energies. The free energy density of the Flory–Huggins model is given by^9−12^
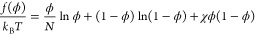
1where *k*_B_ is the Boltzmann constant and *T* is the absolute temperature. In the following, we consider energies relative to the thermal energy and set *k*_B_*T* = 1 to simplify notation. The first two terms on the right-hand side of eq 1 represent the entropic free energy of mixing, while the third term denotes the effective contact energy. Two important quantities can be calculated: the spinodal concentration and binodal concentration. The spinodal is the boundary between locally stable/unstable regions and can be solved exactly, while the binodal separates globally stable/unstable regions, and the system can still be locally stable on the binodal boundary itself. It is also straightforward to generalize eq 1 to include more components or surface tension,^11,14,15^ and over the decades more detailed models have been proposed to include electrostatic interactions^16^ and sticker-spacer behaviors^7,8,17^ or to calculate free energy density from first principles using a field-theoretic approach.^18−21^ It thus appears that the Flory–Huggins theory is an outdated model due to its oversimplifying, mean-field nature, while we note that even then an analytical solution for the binodal concentrations is lacking for this most basic picture of LLPS.

Mathematical formulations of spinodal and binodal concentrations are briefly summarized here. The free energy density becomes locally unstable at *f*″(ϕ) ≤ 0, and consequently the spinodal boundary ϕ^spi^ is defined at the transition point *f*″(ϕ^spi^) = 0. Solving for this condition, we obtain the dense (ϕ_+_^spi^) and dilute phase (ϕ_–_^spi^) spinodal concentrations
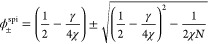
2with , which goes to zero in the symmetric *N* = 1 case. The critical point of LLPS occurs when the dense and dilute phases coincide, corresponding to a critical interaction strength χ_c_ and concentration ϕ_c_:
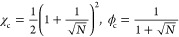
3

Near the critical point with *δχ* ≡ χ – χ_c_ ≈ 0, the spinodal concentrations have the approximate form
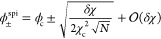
4

Note that in the opposite limit of large *N* or large χ the dilute phase concentration has a power-law scaling
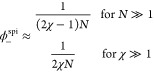
5and these are qualitatively different from the exponential scaling of the dilute phase binodal concentrations, as will be shown using the self-consistent equations.

Next, we outline the steps to obtain binodal concentrations. The binodal concentrations are found by assuming the existence of two distinct phases characterized by polymer volume fractions ϕ_+_, ϕ_–_, and phase volumes *V*_+_, *V*_–_. The equilibrium condition requires minimization of the total energy *F*_tot_ ≡ *V*_+_*f*(ϕ_+_) + *V*_–_*f*(ϕ_–_) subject to total volume and mass conservation conditions *V*_+_ + *V*_–_ = *V*_tot_ and *V*_+_ϕ_+_ + *V*_–_ϕ_–_ = *V*_tot_ϕ_tot_. Using Lagrange minimization, we identify the chemical potential μ(ϕ) ≡ *f*′(ϕ) and osmotic pressure Π(ϕ) ≡ *ϕf*′(ϕ) – *f*(ϕ) as Lagrange multipliers that have to hold the same values in the two compartments:
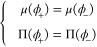
6

Graphically, in a [ϕ,*f*(ϕ)] plot, the μ(ϕ_+_) = μ(ϕ_–_) condition forces the two points describing the coexisting phases to have the same gradient, and Π(ϕ_+_) = Π(ϕ_–_) aligns the two tangent lines to have the same *y*-intercept and as such represent a common tangent construction (Figure 1A). Using the Flory–Huggins free energy eq 1, we have
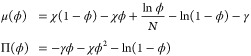
7and the binodal concentrations can be calculated by solving eq 6 with the definitions of eq 7. The objective of this paper is to generate analytical solutions with eq 7. As a first step, an approximate binodal solution near the critical point can be worked out by expanding the free energy around ϕ = ϕ_c_ + *δϕ* and χ = χ_c_ + *δχ* with small *δχ* and *δϕ*. Terms that are constant or linear in *δϕ* drop out of the common tangent construction; terms of order higher than 4 are also truncated. The result is , and for *δχ* > 0 this is a simple Ginzburg–Landau second-order phase transition. The binodal concentrations near the critical point are
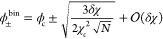
8

**Figure 1:**
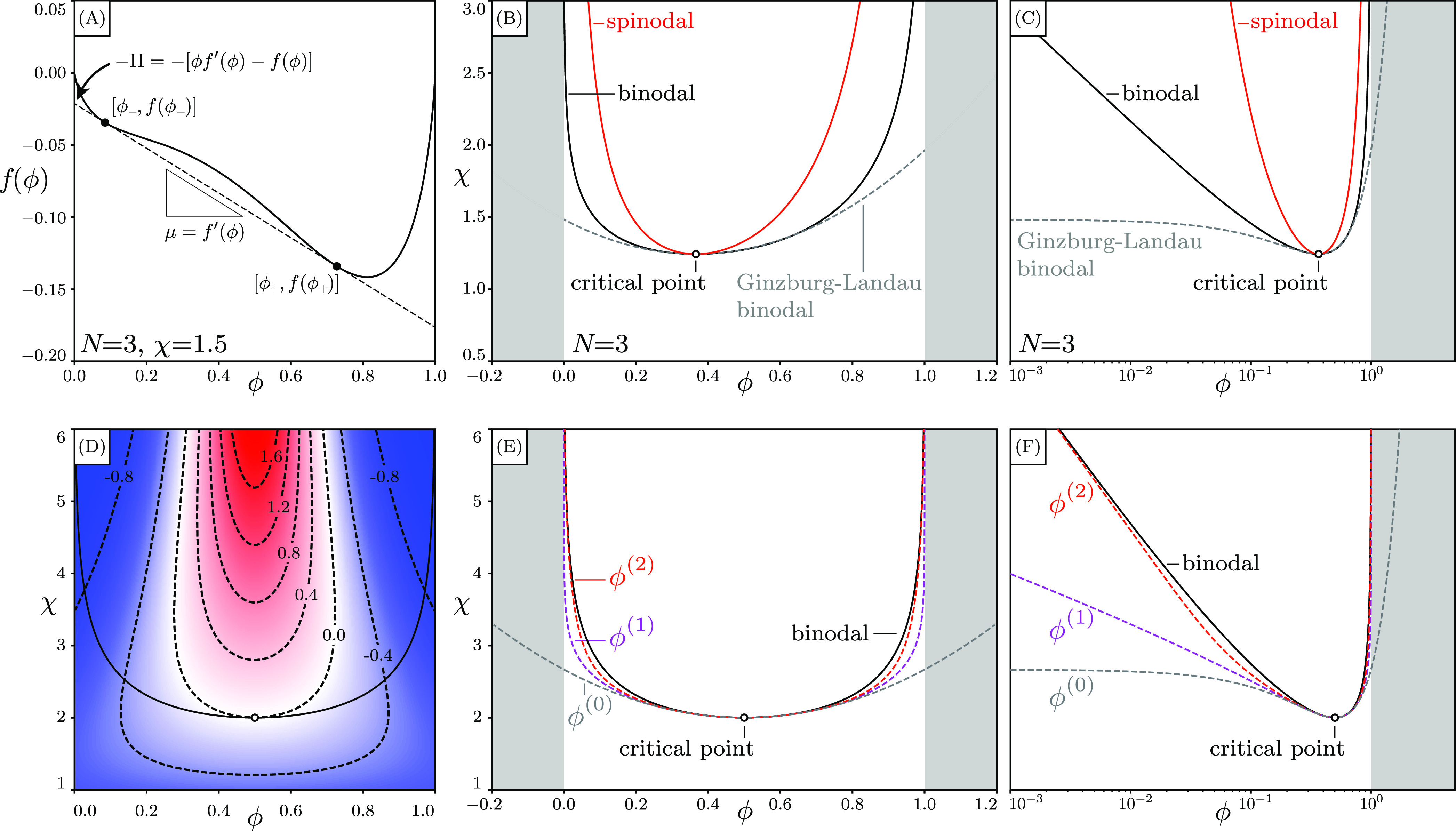
Flory–Huggins model (A–C) and self-consistent solution for the symmetric *N* = 1 case (D–F). (A) Common tangent construction at *N* = 3, χ = 1.5 gives the dense and dilute phase concentrations ϕ_±_. The gradient of the common tangent is the chemical potential μ(ϕ), and the *y*-intercept is −1 times the osmotic pressure Π(ϕ). (B, C) Complete phase diagram of the *N* = 3 system in linear and logarithmic ϕ scales. The binodal is calculated numerically.^11^ Near the critical point, the Ginzburg–Landau binodal approximates the exact binodal well, but at large χ the two quickly diverges and the Ginzburg–Landau solution enters the unphysical range ϕ < 0 and ϕ > 1 (gray zones). (D) Plot of |*g*′(ϕ)| – 1 in ϕ, χ space. The black solid line is binodal, and the hollow circle is the critical point. Dashed lines are contours of constant |*g*′(ϕ)| – 1. The blue region with |*g*′(ϕ)| – 1 < 0 has stable orbits, while the red regions are unstable. (E, F) Comparison between the numerical binodal (black solid line) with self-consistent schemes with 0, 1, and 2 iterations (colored dashed lines).

The Ginzburg–Landau solution describes the binodal near the critical point (Figure 1B,C). We now aim to extend this solution to cover χ far away from χ_c_ through a self-consistent approach, summarized as the following. Suppose we need to solve the equation  with some operator . Instead of solving it directly, we treat  as a discrete map and start with a solution η^(0)^ and apply  iteratively to generate the orbit  with *i* = 1, 2, ... With a suitable form of , the orbit converges to the fixed point lim_*i*→∞_ η^(*i*)^ = η, which then solves the equation . This is the contractive mapping principle.^22,23^ The self-consistent approach has been previously employed to approximate the protein aggregation kinetics curves,^24,25^ and here we show a similar procedure allows efficient and accurate computation of the binodal concentrations.

Starting with the simple case of unit polymer length *N* = 1, the free energy (eq 1) is invariant under a reflection around , i.e., ϕ → 1–ϕ, and the binodal is given by the condition *f*′(ϕ) = 0, leading to . Upon rearrangement, the binodal equation is
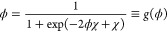
9and we use *g*(ϕ) to define the 1D map
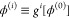
10with the initial guess ϕ^(0)^ to be determined later. The fixed points are the binodal concentrations ϕ_±_^bin^. To study the convergent properties of the map, we expand *g*(ϕ) near the fixed point, writing ϕ_±_^(*i*)^ = ϕ_±_^bin^ + *δϕ*_±_^(*i*)^.^26^ This gives *δϕ*_±_^(*i*+1)^ = *g*′(ϕ_±_^bin^)*δϕ*_±_^(*i*)^, so for a convergent orbit we require |*g*′(ϕ_±_^bin^)| < 1, and quick convergence can be expected for *g*′(ϕ_±_^bin^) ≈ 0. We calculate |*g*′(ϕ)| – 1 in the ϕ, χ space and observe that near the binodal convergence is fast in the high-χ regime with |*g*′(ϕ)| ≈ 0, while it is much slower near criticality χ ≈ χ_c_ = 2 and becomes 0 at exactly the critical point (Figure 1D). We thus need the initial guess to be close to the binodal just at χ ≈ χ_c_ and both the spinodal and approximate binodal may seem to be appropriate choices. It is worth writing down these concentrations near criticality with *N* = 1:  and . Furthermore, we can also obtain the approximate form of the |*g*′(ϕ)| – 1 = 0 contour near χ_c_ = 2 and , which gives exactly . The spinodal thus coincides with the metastable line and is not an appropriate choice, despite it having better behavior than the approximate binodal at large χ: the latter enters the unphysical regions ϕ < 0 and ϕ > 1, while the spinodal is always bound in 0 < ϕ < 1. We thus use the Ginzburg–Landau binodal (eq 8) as the initial guess so
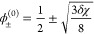
11and the contraction mapping principle allows us to write the solution as
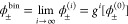
12Good convergence is observed within two iterations (Figure 1E,F). The analytical form at one self-consistent step is
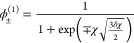
13and this already improves the Ginzburg–Landau solution in linear ϕ scale. However, the large-χ behavior is poorly captured in log scale due to the initial guess entering the unphysical region ϕ < 0 and ϕ > 1, and this problem disappears when a second self-consistent step is performed, which gives
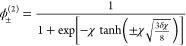
14

These expressions cover both the low and high-χ regime (Figure 1E,F). For large χ ≫ 1, we have , thus giving the scaling law for dilute phase binodal concentration
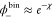
15which is qualitatively different from the polynomial scaling of the spinodal concentration . This exponential scaling has a physical interpretation as the chemical potential in the dilute limit takes the form of μ ≈ ln ϕ. An important implication is thus that the binodal phase separation can occur over a concentration range spanning orders of magnitude, while spinodal decomposition has a much narrower band of concentrations.

Now we extend the *N* = 1 solution to the general case. Using eq 6 with eq 7, the binodal equations are
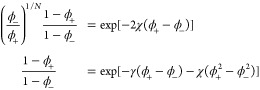
16

Defining the two exponents
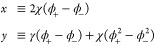
17eqs 16 can be simplified to ϕ_–_/ϕ_+_ = *e*^–*N*(*x*–*y*)^. Substituting this back into the second equation of 16, we get . Solving for ϕ_+_ and then ϕ_–_, we obtain
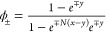
18

Organizing ϕ_+_, ϕ_–_ in vector form:
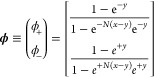
19defining the operator on the right-hand side of eq 19 as ***G***, we have the map
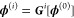
20with the Ginzburg–Landau solution as the initial guess
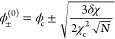
21

A scaling law for the dilute phase at a large interaction strength can be obtained as before. For χ ≫ 1, we make use of the relation ϕ_–_/ϕ_+_ = *e*^–*N*(*x*–*y*)^ and calculate *x*–*y* as
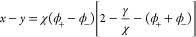
22Observe that , ϕ_+_ + ϕ_–_ < 2 and ϕ_+_ > ϕ_–_, leading to (*x*–*y*) ≫ 1 and ϕ_–_/ϕ_+_ ≈ 0. A trivial “guess solution” is thus ϕ_+_^bin^ ≈ 1 and ϕ_–_^bin^ ≈ 0. Substituting this back into the self-consistent operator gives *e*^–*x*^ ≈ *e*^–2χ^ and *e*^–*y*^ ≈ *e*^–γ–χ^. The self-consistent expression for ϕ_–_ then becomes . This can be further approximated to be
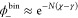
23In the case of *N* = 1, we recover the *e*^–χ^ scaling discussed above. The convergence behavior of the 2D map can also be studied similar to the 1D case. Defining the Jacobian matrix ***J*** as
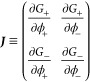
24and writing **ϕ**^(*i*)^ = **ϕ**^bin^ + δ**ϕ**^(*i*)^, we get δ**ϕ**^(*i*+1)^ = ***J***δ**ϕ**^(*i*)^. Stability requires moduli of eigenvalues of ***J*** to be less than 1. Since both the eigenvalues and eigenvectors of ***J*** are in general complex, to better visualize the convergence of the orbit **ϕ**^(*i*)^ we instead promote the discrete map to a continuous flow equation parametrized by *t*: **ϕ**(*t*), with **ϕ̇** = ***G***(**ϕ**)–**ϕ**. The velocity field **ϕ̇** then contains the behavior of the orbit **ϕ**^(*i*)^ in the limit of small time steps, and three fixed points can be identified: one stable fixed point corresponding to the binodal and two unstable fixed points on the ϕ_+_ = ϕ_–_ diagonal (Figure 2A). We observe the orbit **ϕ**^(*i*)^ is indeed convergent (Figure 2B,C).

**Figure 2:**
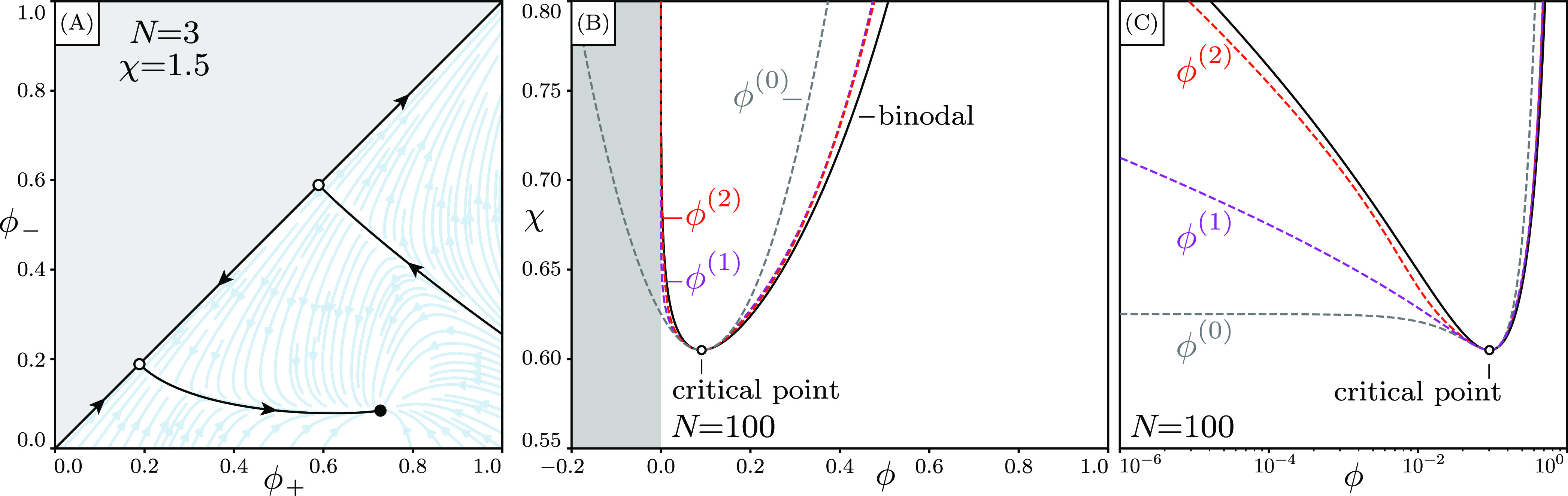
Self-consistent solution for general *N*. (A) Flow field of the continuous map with *N* = 3, χ = 1.5. Solid circle is the stable fixed point corresponding to the binodal, and hollow circles are saddle points. (B, C) Comparison between the numerical binodal (black solid line) with self-consistent schemes with 0, 1, and 2 iterations (colored dashed lines).

To obtain analytical forms for the general binodal, we first simplify notations by defining
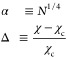
25and express all other parameters in terms of α, Δ. This allows us to write
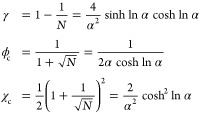
26The Ginzburg–Landau solution (eq 21) then takes the simple form
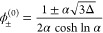
27and *x*, *y* as defined in eq 17 are now
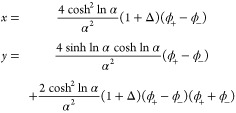
28

Direct substitution gives
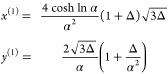
29so at one self-consistent step we have
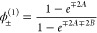
30where
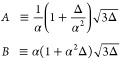
31*A* and *B* are related through the transformation  as  and vice versa. The large-χ behavior is incompletely captured in log scale (Figure 2C). We thus again calculate the second-order self-consistent solution. At second order, we substitute eq 30 into eq 28 and obtain
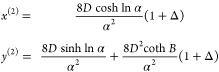
32where
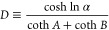
33and *D* is invariant under the transformation . The second-order expression is then
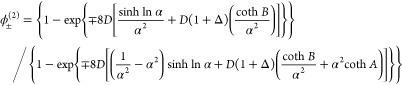
34Notice again that the denominator is invariant under the transformation . The second-order analytical form approximates the exact binodal to a high degree (Figure 2B,C) even at the large-*N* regime.

Although the self-consistent solutions are exact near critical points and convergent at large χ, the convergence is slow in the transition region. Here we show that we can improve the maps by performing a first-order expansion of the self-consistent operator. Starting from a general self-consistent equation  and an initial guess η^(0)^, we want to find a step *δη* such that the next guess η^(1)^ ≡ η^(0)^ + *δη* solves the self-consistent equation to first order. We thus write . Expanding  to first order, and solving for *δη* we obtain
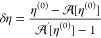
35so the next best guess is
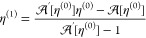
36

The above results can readily be applied to improve the maps *g*(ϕ) and ***G***(**ϕ**). In the *N* = 1 case, we define the new map *h*(ϕ) as
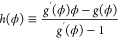
37and it reduces to the original map when *g*′(ϕ) = 0. We thus define the new orbit ϕ_*h*_^(*i*)^ ≡ *h*^*i*^[ ϕ^(0^]. The convergent property can be studied by expanding the above with ϕ_*h*_^(*i*)^ = ϕ* + *δϕ*_*h*_^(*i*)^, and we arrive at
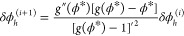
38and near the fixed point the numerator approaches 0, so the convergence is rapid (Figure 3A).

**Figure 3:**
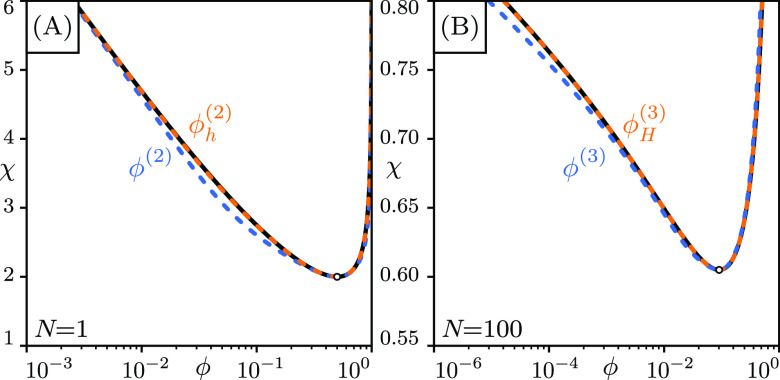
Improved self-consistent solutions from self-consistent expansion (orange dashed lines) converge to the numerical solution (black solid lines) more quickly than the original ones (blue dashed lines), in both the (A) symmetric *N* = 1 case and (B) the general *N* case. The numerical binodal virtually overlaps with the improved solutions.

In the case of general *N*, we similarly obtain
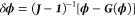
39with **1** a 2 by 2 identity matrix. The improved operator is
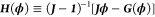
40

Good agreement with numerical results is achieved for the new orbit **ϕ**_*H*_^(*i*)^ ≡ ***H***^*i*^[**ϕ**^(0)^] within three iterations for a large *N* = 100 (Figure 3B).

The self-consistent solution allows efficient computation of binodal concentrations, and we use it to fit experimental LLPS data and extract the interaction parameters. In the following fitting, we use eq 34 to compute the binodal concentrations. Binodal concentrations for the prion-like low-complexity domain from isoform A of human hnRNPA1 (A1-LCD) were measured in ref (27) (137 amino acid residues), and three series of A1-LCD variants are fitted here. The first series involves aromatic residues tyrosine (Y) and phenylalanine (F); the second series involves nonequivalent polar spacers glycine (G) and serine (S); and the third series involves ionic residues aspartic acid (D), arginine (R), and lysine (K). The wild type (WT) A1-LCD binodal was also measured. During fitting, the chain length *N* is set as a global fitting parameter. The interaction parameter χ has the form , and we fit *Δϵ* for each variant. To convert concentrations to volume fractions, we use a protein density of 1.35 g/cm^3^ and average molecular weight of 13.1 kDa to obtain the conversion ratio from concentration *c* (M) to volume fraction ϕ as . The fitting results give the effective chain length *N* = 158.6, larger than 137, the number of residues. These results can appear counterintuitive, as past studies have postulated an effective protein segment length larger than the size of an amino acid,^28,29^ so the effective *N* should be smaller than the number of residues. The discrepancy arises from a subtle difference in the definition of *N* in the Flory–Huggins picture as compared to the polymer picture: here the fitted *N* represents the number of lattice sites occupied by each solute and does not depend on its polymeric nature. As a result, the same *N* can be defined for nonpolymer solutes such as micelle clusters, and thus the *N* estimated here should not in any way relate to the effective segment length of the polymer. In the present case, the lattice site volume is determined by the underlying medium, i.e., water, so the effective *N* will be larger than the number of residues owing to the larger size of amino acids compared to water molecules. The ratio  then represents the average number of lattice sites occupied by one residue. The fitted *Δϵ* values represent the site-to-site contact energy, and a larger *Δϵ* indicates a stronger attraction between proteins. To highlight the difference across variants, we first calculate the protein-to-protein contact energy *E* ≡ – *NΔϵ* and define the deviation from WT as *ΔE*_variant_ ≡ *E*_variant_ – *E*_WT_. Fitted curves are plotted in Figure 4, and *ΔE* results are listed in Table 1. Each variant series then allows quantitative interpretation of impacts of different residues on LLPS propensity. In principle, the effective interaction energy *E*_variant_ is a function of the whole amino acid sequence that depends on both the composition and arrangement of individual residues. For example, relating the detailed sequence information to the effective interaction energy has been achieved for polyelectrolytes through the sequence charge decoration (SCD) parameter^30^ with pairwise binding constants and second virial coefficient expressed in terms of SCD.^31^ Finding this function for a generic protein can be hard, although machine-learning techniques could potentially be used with enough protein sequences and corresponding binodal data. In the present case, only limited data are available, so we assume a simple, linear functional form of *E*_variant_ to illustrate the utility of the self-consistent solution. To this end, we simply assume *E*_variant_ = *E*_0_ + ∑_*i*_*n*_*i*_*ΔE*_*i*_ with *E*_0_ a constant and *n*_*i*_, *ΔE*_*i*_ the number and effective contribution of the amino acid residue *i*. This then allows us to construct linear simultaneous equations from the fitted values (Table 1) and quantify the energetic contribution of individual residues.

**Figure 4:**
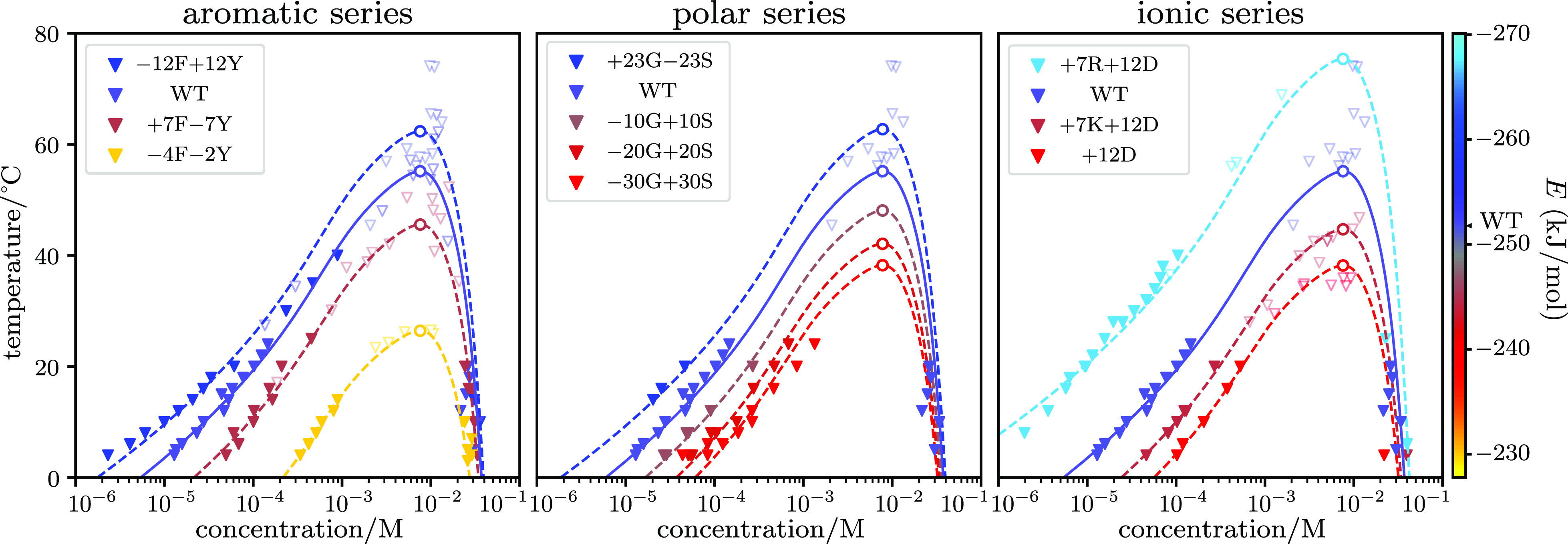
Flory–Huggins fit of binodal data from ref (27), fitted using eq 34. A constant *N* is maintained across all variants. Solid triangle markers are dilute and dense phase concentration measurements, and light hollow triangle markers are estimates of the critical point from cloud point measurements. Dashed lines are the best-fit curves, and hollow circles are critical points. The WT binodal is the same in all three plots and is plotted in the solid line. Colors of the plot represent the *E* ≡ −*NΔϵ* values of the variant. The ±*nX* in variant names indicate *n* of *X* residues are added (*+*) or removed (−) from WT.

**Table 1:** Fitting Results for the A1-LCD Data^a^

*N* = 158.6 *E*_WT_ = −251.9 kJ/mol					
aromatic series		polar series		ionic series	
variant	*ΔE* (kJ/mol)	variant	*ΔE* (kJ/mol)	variant	*ΔE* (kJ/mol)
–12F +12Y	–5.5	+23G −23S	–5.8	+7R +12D	–15.5
–7F −7Y	+7.4	–10G +10S	+5.4	+7K +12D	+8.0
–4F −2Y	+22.0	–20G +20S	+10.0	+12D	+13.0
		–30G +30S	13.0		

a Differences in *ΔE* across variants allow contributions of individual residues to be inferred.

Results from the aromatic series indicate that tyrosine is a stronger sticker than phenylalanine, in line with previous observations.^27^ We further infer the individual contribution of each Tyr and Phe residue, *ΔE*_Tyr_ and *ΔE*_Phe_, using values from Table 1:
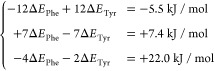
41The first two equations give *ΔE*_Phe_–*ΔE*_Tyr_ = 0.8 ± 0.3 kJ/mol, with the error arising from the difference in the measured per-residue energy change. This can then be combined with the third equation to give *ΔE*_Tyr_ = −4.2 ± 0.2 kJ/mol and *ΔE*_Phe_ = −3.4 ± 0.1 kJ/mol. Both Tyr and Phe are thus stickers with Tyr stronger than Phe.

For the polar series, we perform similar calculations and extract the difference *ΔE*_Gly_ – *ΔE*_Ser_ = −0.43 ± 0.11 kJ/mol, indicating the destabilizing effect of serine residues. This can be understood as the OH group in Ser forming favorable interactions with water, thus destabilizing the condensate.

The ionic series data are harder to interpret since the overall protein charge can have a non-monotonic effect on LLPS propensity.^27^ We can however still compare the +7R +12D and +7K +12D variants since they have the same overall charge. The energy difference between arginine and lysine is *ΔE*_Arg_ – *ΔE*_Lys_ = −3.4 kJ/mol, indicating stronger sticker behavior for arginine. This can arise due to the electron delocalization in the guanidinium group and higher charge–charge contact efficiency with other charged residues, or stronger cation-π interaction from coplanar packing.^32,33^ Furthermore, it should be noted that despite the simple linear functional form of *E*_variant_ assumed here, the resulting energy difference between Arg and Lys is in line with atomistic simulation results: using the Kim-Hummer model,^34,35^ the difference in average residue–residue pair interaction involving either Arg or Lys is estimated to be (1.48–2.22)*k*_B_*T* = −0.74*k*_B_*T* ≈ −1.84 kJ/mol.^32^ This is roughly half of −3.4 kJ/mol as estimated from LLPS data, and a probable reason is that one Arg or Lys residue might be involved in more than one residue–residue contact, giving a higher overall contribution to the contact energy.

To conclude, we have developed a self-consistent solution for the binodal concentration of the two-component Flory–Huggins phase-separating system. The proposed self-consistent operators shed light on the scaling behavior of the dilute phase binodal, which is qualitatively different from the scaling of the spinodal and explains why LLPS of proteins occurs over a concentration range spanning several orders of magnitude. Using the well-known Ginzburg–Landau binodal approximate solution as the initial guess, the self-consistent solution achieves numerical accuracy within two to three iterations and allows highly efficient fitting of experimental binodal data. Explicit analytical forms of the binodal concentrations are also proposed to approximate the binodal. Using the developed solution, we fitted experimental data measured for variants of the A1-LCD protein and extracted effective interaction energies, which can be used to further decode the impact of individual amino acid residues on LLPS. Our analytical solution to the Flory–Huggins model thus allows systematic investigation of sequence grammar of LLPS-prone proteins and, with sufficient experimental data, can lead to development of a wholistic framework for predicting LLPS propensity from sequence information.

## Supporting Information Available

Transparent Peer Review report available (PDF)

## Notes

Code availability: A python library containing the self-consistent maps and auxiliary functions can be found at: https://github.com/KnowlesLab-Cambridge/FloryHuggins.
