# Supplementary material for: Analytical Solution to the Flory–Huggins Model
Source: J Phys Chem Lett. 2022 Aug 17;13(33):7853–60. doi: 10.1021/acs.jpclett.2c01986 (PMC9421911; doi:10.1021/acs.jpclett.2c01986)
Supplement: Supplementary file 1 — jz2c01986_si_001.pdf [file jz2c01986_si_001.pdf]

jz-2022-01986b.R1

Name: Peer Review Information for "Analytical Solution to the Flory-Huggins Model"

First Round of Reviewer Comments

Reviewer: 1

Comments to the Author

The Flory-Huggins solution theory is central to our understanding of phase transitions in soft condensed matter. Recently, it has received renewed interest due to the discovery of polymer phase separation in biological systems. Despite the importance of the model, the analytic intractability of the double-tangent construction means that calculation of the binodal (which is the experimental observable in most cases) requires cumbersome numerical methods, limiting widespread use of the model in data fitting. This work addresses that limitation using a clever self-consistent formalism to iteratively achieve analytical expressions of increasing accuracy. I found the math relatively easy to follow and the authors are to be commended by building intuition but ramping up complexity in the presentation. The widespread use of the Flory-Huggins model means that this work is worthy of rapid dissemination and make a good Letter. I have only minor comments for the authors.

The lines in figure 3 are very difficult to distinguish (a good problem to have!) It might be easier to see if the numerical line was thicker than the self-consistent solutions and a more contrasting color was chosen for the line that overlaps with it (for example, thin red dashes overlapping with a thick black line).

There appears to be an error in the aromatic series because they have three equations for two variables: Ephe and Etyr. Perhaps one of them is mislabeled tryptophan? Or are they using a fitting procedure rather than solving a system of equations?

Finally, the evaluation of sticker strengths could use a few sentences of clarification because they are comparing a heteropolymer to a homopolymer theory. Therefore, their results are giving the average chi a given residue interacting with the mean field of all residues. This has two implications for their results. First, the residue-specific chi value they obtain depends on the sequence of the rest of the molecule. Second, mutating residues also changes the mean field felt by the new residues. This could be a significant effect when they are replacing >10% of the residues in their mutation series.

Reviewer: 2

Comments to the Author

Referee Report on jz-2022-01986b: Qian, Michaels & Knowles

The new formulation reported in this manuscript is elegant. It constitutes a major advance in polymer solution theory. The method developed by the authors is timely because it can facilitate efficient comparisons of experimental liquid-liquid phase separation (LLPS) data with Flory-Huggins (FH) theory. Nonetheless, there is still room for improvement in the analysis and presentation of the authors' results, especially with regard to the physical implications of the fitting parameters in Sec. VI, and, to a lesser extent, the stated scope of the theory. Accordingly, the manuscript should be revised to address the following before acceptance for publication:

1. *Scope and limitation of FH:* FH is a mean-field theory that accounts for composition effects but not full sequence effects on polymer interactions. In other words, different sequences with the same composition produce the same behavior in the FH description. With this in mind, statements regarding the utility of the authors' method to the study of "sequence grammar" (in the Abstract and in the Conclusion Sec. VII) should be qualified so that readers who are less familiar with the field would understand precisely that only the composition aspect of "sequence grammar" is accounted for by the theory in this manuscript.

2. *Physical implication of the fitted N value:* In Sec. VI, chain length N is used as a fitting parameter, yielding the result that each lattice site in the FH lattice represents 137/158.6 = 0.86 amino acid residue. Notably, this factor is significantly different from a previously proposed correspondence of 1 FH lattice ~ 1.4 residues based on a physically plausible isodiametric argument [Dill, Biochemistry 24:1501-1509 (1985)]. Consistently, since chains on FH are fully flexible (similar to random flights in this regard) but polypeptide chains have persistence lengths longer than the C_alpha-C_alpha virtual bond length of 3.8 angstrom, one expects from this physical consideration that this factor should be larger than unity, not smaller than one. Indeed, a recent estimation based on explicit-chain simulations yielded a factor of 6.46/3.8 = 1.7 [Song et al., J Phys Chem 125:6451-6478 (2021); see discussion immediately below Eq.30 of this reference] which is even larger than the isodiametric-based factor of 1.4. In this context, the difference between the authors' <1 fitted chain length ratio and these previous >1 estimates should be noted in the revised manuscript with the references included. Possible mathematical/physical reasons for the authors' smaller-than-unity FH-lattice/residue ratio should be discussed accordingly.

3. *Effects of Arg vs Lys on LLPS propensity:* On page 7 (P.7), subsection on Arginine vs Lysine, it is stated that the observed trend of arginine having more favorable interactions than lysine may be caused by "electron delocalization" etc. In this regard, it should be noted that pi-interactions have also been proposed as a likely reason for more favorable of Arg-related interactions [see, e.g., Das et al., PNAS 117:28795-28805 (2020)]. Also emphasized in this reference is that the difference between Arg and Lys may be rationalized by the protein folded structure-based "KH" statistical potential used by Dignon et al. for LLPS simulations [PLoS Comput Biol 14:e1005941 (2018)] (though KH does not account for the difference in LLPS effects of Phe vs Tyr). In the analysis of Das et al., the average residue-residue contact energy for Arg is calculated to be -2.22k_BT (averaged over 20 amino acid residue types), whereas the correspondence average for Lys is -1.48k_BT, netting an Arg-Lys difference of -0.74 k_BT in average residue-residue contact energy, which amounts to -1.85 kJ/mol at T = 300K. It is noteworthy that this number is about half of the Delta E_Arg - Delta E_Lys = -3.4 kJ/mol reported in Sec. VI of the manuscript. Since the chi parameter (which is the basis for the authors' fitted Delta E values) contains a factor z (lattice coordination number, Sec. II), meaning that most likely Delta E accounts for more than one (at least about two or more) residue-residue contacts, one may surmise that as far as the effect of Arg vs Lys on LLPS is concerned, the authors' fitted Delta E values are consistent with the KH potential. This

feature (and pi-interactions being one of the possible physical origins) should be discussed briefly in the revised version to place the present work in a more comprehensive physical context.

4. *Typos and suggested improvements for clarity:*

P.1, 5th line below Eq.1: “entropic energy” should be “entropic free energy”

P.3, 2nd line in Sec. 3: It would be helpful for readers to specify “symmetrical” by stating that the system is symmetric with respect to (phi <--> 1 – phi) interchange.

P.4, Eqs. 17-22: In the derivation of the scaling relation, it would be helpful to state (possibly with a very brief explanation) that x – y > 0 for large positive chi (i.e., chi >> gamma).

P.4, Eq.25: A factor of ½ is missing on the right hand side of the middle equation for phi_c (i.e., a factor of 2 should be in the denominator, as in Eq.26).

P.6, 13th line of the “Fitting results” subsection: “owning” should be “owing”?

Author's Response to Peer Review Comments:

We thank the reviewers for the helpful and constructive feedbacks. All points raised have been addressed and detailed responses are included in the PDF file attached.

Response to reviewer comments

I. REVIEWER 1 COMMENTS

The Flory-Huggins solution theory is central to our understanding of phase transitions in soft condensed matter. Recently, it has received renewed interest due to the discovery of polymer phase separation in biological systems. Despite the importance of the model, the analytic intractability of the double-tangent construction means that calculation of the binodal (which is the experimental observable in most cases) requires cumbersome numerical methods, limiting widespread use of the model in data fitting. This work addresses that limitation using a clever self-consistent formalism to iteratively achieve analytical expressions of increasing accuracy. I found the math relatively easy to follow and the authors are to be commended by building intuition but ramping up complexity in the presentation. The widespread use of the Flory-Huggins model means that this work is worthy of rapid dissemination and make a good Letter. I have only minor comments for the authors.

We thank the reviewer for the encouraging feedback.

The lines in figure 3 are very difficult to distinguish (a good problem to have!) It might be easier to see if the numerical line was thicker than the self-consistent solutions and a more contrasting color was chosen for the line that overlaps with it (for example, thin red dashes overlapping with a thick black line).

This is a very helpful suggestion and we have implemented the change.

There appears to be an error in the aromatic series because they have three equations for two variables: Ephe and Etyr. Perhaps one of them is mislabeled tryptophan? Or are they using a fitting procedure rather than solving a system of equations?

Here we are solving the two equations in (41) individually and combine them with an error estimate. We have now changed the text to explicitly explain this.

Finally, the evaluation of sticker strengths could use a few sentences of clarification because they are comparing a heteropolymer to a homopolymer theory. Therefore, their results are giving the average chi a given residue interacting with the mean field of all residues. This has two implications for their results. First, the residue-specific chi value they obtain depends on the sequence of the rest of the molecule. Second, mutating residues also changes the mean field felt by the new residues. This could be a significant effect when they are replacing >10% of the residues in their mutation series.

Indeed, the original explanation is lacking clarity about the interpretation of χ in the context of realistic systems. We propose that χ should be a function of the whole sequence, i.e. it depends on both the composition and the arrangement of residues. Machine learning techniques can be used to approximate such a function with enough data and here we use a simple linear form to interpret the available dataset. This discussion has been added in section VI.

II. REVIEWER 2 COMMENTS

The new formulation reported in this manuscript is elegant. It constitutes a major advance in polymer solution theory. The method developed by the authors is timely because it can facilitate efficient comparisons of experimental liquid-liquid phase separation (LLPS) data with Flory-Huggins (FH) theory. Nonetheless, there is still room for improvement in the analysis and presentation of the authors' results, especially with regard to the physical implications of the fitting parameters in Sec. VI, and, to a lesser extent, the stated scope of the theory. Accordingly, the manuscript should be revised to address the following before acceptance for publication:

We thank the reviewer for the constructive feedback and have added more discussions on practical aspects of the self-consistent solution. Overall we think the suggestions are really valuable and are greatly improving the quality of the paper.

1. Scope and limitation of FH: FH is a mean-field theory that accounts for composition effects but not full sequence effects on polymer interactions. In other words, different sequences with the same composition produce the same behavior in the FH description. With this in mind, statements regarding the utility of the authors' method to the study of "sequence grammar" (in the Abstract and in the Conclusion Sec. VII) should be qualified so that readers who are less familiar with the field would understand precisely that only the composition aspect of "sequence grammar" is accounted for by the theory in this manuscript.

This is a very interesting point! The mean-field nature of the Flory-Huggins theory actually does not prevent us from incorporating the sequence information into the theory. The reason is that given an amino acid sequence, we can construct a Flory χ parameter based on both the composition and sequence (this has been achieved with the Sequence Charge Decoration parameter in polyelectrolyte coacervation and will certainly be possible if sufficient data is available and a machine-learning approach can be used). The approach presented in section VI is a simple demonstration of the utility of the derived solution, where it is reasonable to assume a purely composition-dependent χ given the limited amount of data, and we have added more discussions on this aspect in the main text.

2. Physical implication of the fitted N value: In Sec. VI, chain length N is used as a fitting parameter, yielding the result that each lattice site in the FH lattice represents 137/158.6 = 0.86 amino acid residue. Notably, this factor is significantly different from a previously proposed correspondence of 1 FH lattice $\sim$ 1.4 residues based on a physically plausible isodiametric argument [Dill, Biochemistry 24:1501-1509 (1985)]. Consistently, since chains on FH are fully flexible (similar to random flights in this regard) but polypeptide chains have persistence lengths longer than the C_alpha-C_alpha virtual bond length of 3.8 angstrom, one expects from this physical consideration that this factor should be larger than unity, not smaller than one. Indeed, a recent estimation based on explicit-chain simulations yielded a factor of 6.46/3.8 = 1.7 [Song et al., J Phys Chem 125:6451-6478 (2021); see discussion immediately below Eq.30 of this reference] which is even larger than the isodiametric-based factor of 1.4. In this context, the difference between the authors' <1 fitted chain length ratio and these previous >1 estimates should be noted in the revised manuscript with the references included. Possible mathematical/physical reasons for the authors' smaller-than-unity FH-lattice/residue ratio should be discussed accordingly.

This is another good point! There is a subtlety involving the physical meaning of the parameter N: in the Flory-Huggins theory, N represents a reduction in translational entropy due to a solute size larger than the lattice size, and it is independent of the polymeric nature of the solute. As such, N can be defined in the Flory-Huggins sense for solutes that are non-polymeric, for example a micelle or a crystal nucleus as in the classical nucleation theory, while the concept of N as the effective segment number of a polymer can be defined only for polymers. We have made this clearer in the manuscript right after the N estimation.

3. Effects of Arg vs Lys on LLPS propensity: On page 7 (P.7), subsection on Arginine vs Lysine, it is stated that the observed trend of arginine having more favorable interactions than lysine may be caused by "electron delocalization" etc. In this regard, it should be noted that pi-interactions have also been proposed as a likely reason for more favorable of Arg-related interactions [see, e.g., Das et al., PNAS 117:28795-28805 (2020)]. Also emphasized in this reference is that the difference between Arg and Lys may be rationalized by the protein folded structure-based "KH" statistical potential used by Dignon et al. for LLPS simulations [PLoS Comput Biol 14:e1005941 (2018)] (though KH does not account for the difference in LLPS effects of Phe vs Tyr). In the analysis of Das et al., the average residue-residue contact energy for Arg is calculated to be -2.22kBT (averaged over 20 amino acid residue types), whereas the correspondence average for Lys is -1.48kBT, netting an Arg-Lys difference of -0.74 kBT in average residue-residue contact energy, which amounts to -1.85 kJ/mol at T = 300K. It is noteworthy that this number is about half of the Delta E_Arg - Delta E_Lys = -3.4 kJ/mol reported in Sec. VI of the manuscript. Since the chi parameter (which is the basis for the authors' fitted Delta E values) contains a factor z (lattice coordination number, Sec. II), meaning that most likely Delta E accounts for more than one (at least about two or more) residue-residue contacts, one may surmise that as far as the effect of Arg vs Lys on LLPS is concerned, the authors' fitted Delta E values are consistent with the KH potential. This feature (and pi-interactions being one of the possible physical origins) should be discussed briefly in the revised version to place the present work in a more comprehensive physical context.

This is a great suggestion and we have amended the manuscript accordingly.

4. Typos and suggested improvements for clarity:

P.1, 5th line below Eq.1: "entropic energy" should be "entropic free energy"

P.3, 2nd line in Sec. 3: It would be helpful for readers to specify "symmetrical" by stating that the system is

symmetric with respect to (phi $<--->$ 1 – phi) interchange.

P.4, Eqs. 17-22: In the derivation of the scaling relation, it would be helpful to state (possibly with a very brief explanation) that x - y $>$ 0 for large positive chi (i.e., chi $\gg$ gamma).

P.4, Eq.25: A factor of 1/2 is missing on the right hand side of the middle equation for phi_c (i.e., a factor of 2 should be in the denominator, as in Eq.26).

P.6, 13th line of the "Fitting results" subsection: "owning" should be "owing"?

All of the above have been amended.
